# Serum Amyloid A (SAA) induces transcription affecting inflammation

**DOI:** 10.1371/journal.pone.0341858

**Published:** 2026-02-19

**Authors:** George H. Sack, Joseph Yun, C. Conover Talbot, Jasmeet Sethi

**Affiliations:** 1 Departments of Physiology, Pharmacology and Therapeutics and Medicine, Johns Hopkins University School of Medicine, Baltimore, Maryland, United States of America; 2 Department of Biomedical Engineering, Johns Hopkins University, Baltimore, Maryland, United States of America; 3 Single Cell and Transcriptomics Core, Johns Hopkins University School of Medicine, Baltimore, Maryland, United States of America; Universidade de Sao Paulo Instituto de Quimica, BRAZIL

## Abstract

The 104 aa protein Serum Amyloid A (SAA) is a prominent member of the acute phase response (APR) a remarkably conserved and stereotyped set of serum protein changes associated with inflammation and other stimuli. N-terminal fragments of SAA can form fibrils that accumulate in organs (where they are called “amyloidosis”). Recent reports have shown SAA involvement in inflammation, particularly with macrophages, consistent with its role as a “biomarker.” In contrast to this perception of passivity, we report that exposure to both N-terminal decapeptides and intact SAA monomers can induce multiple transcripts in both enteroids and HEK293 cells. The spectrum of transcripts prominently includes proteins related to inflammation and NF-κB control, specifically NFKB1A, TNFA1P3 and IER3. SAA thus can act directly through specific transcription to alter cellular physiology in cells outside the monocyte/macrophage lineage with direct effects on inflammation, likely helping explain its remarkable evolutionary conservation as part of primordial defense.

## Introduction

Serum amyloid A (SAA) proteins constitute a small and closely-related family. They are remarkably conserved throughout primate evolution with related molecules also seen in non-primates [1]. Human SAA1 (104 aa) is one of the most prominent serum proteins in the stereotyped mammalian acute phase response (APR) during which it shares a temporal relationship with levels of C-reactive protein [CRP]. Serum levels of SAA1 can rise 500–1000 fold within 24 hours as part of the APR pattern and then return to baseline levels [2]. The liver is the main source of SAA1; in the mouse as much as 5% of hepatic mRNA encoding SAA can be found at the peak of the APR response.

Despite the dramatic change in serum levels of SAA1 and its striking evolutionary conservation a clear biologic “role” for SAA1 in physiology has not been identified. Chronically elevated serum SAA1 levels (*e.g.,* in chronic inflammatory conditions) can be associated with deposition of highly-ordered fibrils comprising its N-terminal 60–70 aa in various organs where they are known as “secondary amyloid.” Ultimately, these otherwise inert fibrillar deposits can cause organ failure [1] and their tinctorial properties led to the introduction of the term “amyloid” (*Lat* “starch-like”).

Elevated serum levels of SAA1 are associated with many pathologic conditions including chronic infections, malignancies, atherosclerotic change(s), genetic disorders (*e.g.,* Familial Mediterranean fever and others), trauma, and rheumatologic disorders [2]. The three-dimensional structure of the SAA1 monomer is known [3]. It is lipophilic and interacts with lipids (particularly HDL) and membranes [4].

The dramatic elevation of SAA mRNA levels in mouse liver during the APR permitted Morrow *et al.* [5] to isolate and clone the responsible gene sequence. Human counterpart(s) were isolated based on the murine sequence [6]. The “SAA family” constitutes several contiguous small, closely-related genes on human chromosome 11 and murine chromosome 7.

Considerable understanding of control of SAA transcription has established proximal effect(s) of IL-1, IL-6, and TNF [7–9]. However, downregulation of transcription (which accompanies resolution of the APR), remains less well-studied. A persisting question has been whether the latter reflects simple withdrawal of the stimulus(i). An alternative possibility, proposed earlier [1], is that SAA1, with its impressive conservation and dynamic changes in serum levels, may, by itself, directly modulate cell metabolism. Multiple cell surface receptors have been proposed for mediating SAA1 interactions in different systems [1], but no single interaction site has been identified. Galán-Díez *et al.* [10] reported participation by SAA in a feedback loop interacting with the aryl hydrocarbon receptor (AHR) to stimulate mRNA levels for indoleamine 2,3-dioxygenase 1 (*IDO1*) in acute myeloid leukemia. In the mouse ileum, SAA added to dendritic cells and naïve CD4^+^T cells induced Th17 cell differentiation and IL-17 secretion although the precise details were not identified [11].

SAA is an innate opsonin for Gram-negative bacteria [12,13] and is particularly important for colonization of human macrophages by *Mycobacterium tuberculosis* (*Mtb*) [14]. Kawka *et al*. found similar prominent levels of transcription of pro-inflammatory cytokines in macrophages exposed to either SAA-opsonized *Mtb* or SAA alone [15]. Gaiser *et al*. found 64 upregulated genes in macrophages exposed to SAA in a pattern consistent with “classical” macrophage activation. We have sought to address direct effects of SAA1 on broader aspects of cellular metabolism by examining transcription patterns following SAA exposure in tissue culture systems. Earlier reports [16–18] proposed that SAA exposure to bovine intestinal cells helped neonatal protection and we began with study of transcription response(s) in enteroids.

## Materials and methods

### N-terminal oligomer studies using enteroids

Decapeptides corresponding to the N-terminus of bovine mSAA [12] (sequence #1 M-W-G-T-F-L-K-E-A-G) and a scrambled sequence (#2 G-K-F-A-W-E-G-M-T-L) were the kind gift of Dr. Thomas McDonald. Purity was verified by mass spectroscopy (parent ion 1139.36). Aliquots were prepared at 100 µg/ml in phosphate buffered saline (PBS).

A preliminary study used human colonoid monolayers derived from left/ascending colon biopsies as part of the Johns Hopkins GI Organoid Biobank (specific line identifier “107C”) managed by Dr. Jennifer Foulk-Abel. The organoids had been stripped of personally identifiable information. To study adherence of enterohemorrhagic *E. coli* the enteroids (1-07C P19 111021) were grown in transwells using NDM media (the kind gift of Drs. Mark Donowitz and Jennifer Foulk-Abel). Bacterial colony counts were made after 1 hour pretreatment with decapeptides; adherence showed a > 1 log increase for cells exposed to decapeptide #1 and no change for cells treated with decapeptide #2. These studies were not extended but helped guide subsequent approaches, in particular our use of decapeptide #1 (corresponding to mSAA).

Further study also used the enteroids (107C P19 111021) grown in transwells as noted above. The solution containing decapeptide 1 was added to both the top (10µl [8.8 pmole]/100µl) and bottom (60µl [53 pmole]/600µl) of the transwells. Similar volumes of PBS were added to control wells. Following exposure to the decapeptide or control mix, incubation continued for up to 4 hrs.

For harvest, the medium was removed and the cells were washed with 0.5 ml of PBS at 4ᵒ. RNA isolation from both the upper and lower layers of the transwells was performed using the PureLink® RNA MiniKit beginning with 300 µl of “Lysis Buffer” in which the cells were triturated and transferred to a fresh tube, 300µl of 70% ethanol was added and the tubes were placed at −20ᵒ C. overnight. Continuing the PureLink® protocol, the final sample volume was 50µl. The samples were frozen at −20ᵒ and transferred to the laboratory for transcriptional characterization.

### N-terminal decapeptide studies using HEK293 cells

HEK293 cells (293 (HEK293) ATCC CRL-1573^TM^), recognized as female but with personal information deidentified, were seeded at 1.7 X 10^4^ cells per well and grown to confluent monolayers on a poly-D-lysine coated 24-well plate. Adjacent wells received 70 µl of decapeptide 1 (100 µg [62 p mole]/ml in HOH) or BSA solution alone. Incubation continued at 37ᵒ as noted. Harvesting used the protocol noted above.

### Full-length SAA studies using HEK293 cells

Encouraged by the results using decapeptide 1 we next studied response to full-length SAA1 protein. HEK293 cells were grown to confluent monolayers on a poly-D-Lysine coated 24-well plate using MEM-10 with penicillin and streptomycin. Monolayers were washed with 0.5 ml PBS and fresh MEM-10 was added. Recombinant full-length human SAA protein (PEPCO – AF-300–53) was dissolved in HOH (with 0.05% BSA) to a final concentration of 1 µg/µl (85 pmole/ml). Wells received either 50µl of the SAA (4.3 mµmole) solution or 50µl of HOH + 0.05% BSA. Incubation at 37ᵒC continued for the times noted until harvest when the medium was removed and the wells were washed with 0.5 ml PBS. RNA preparations used the Purelink® protocol described above with a final sample volume of 50 µl.

### Library construction and analysis

Libraries were prepared using Illumina’s Stranded Total RNA Prep Ligation with Ribo-Zero Plus kit using 100ng total RNA input amount. RNA quantitation was done using Nanodrop and RNA quality was checked on an Agilent Fragment Analyzer using High Sensitivity total RNA assay. Library concentrations were measured with Qubit; quality was checked on an Agilent Fragment Analyzer using the High Sensitivity Large Fragment method. Libraries were normalized to 5nM for pooling and the library pool concentration was verified by both Fragment Analyzer assay and qPCR. A further library dilution was made @ 4nM in 50ul volume for sequencing on NovaSeq X Plus.

Quality checking was first done using using Fastqc (0.11.9). Reads were aligned to the hg38 human reference genome using STAR aligner (2.7.10a). Counts were extracted using htseq (2.0.5). Differential RNA expression analysis was performed on R (4.4.0) using DESeq2 (1.44.0). For the enteroid dataset, p-values were computed using the Wald test; for the full-length SAA dataset, p-values were computed using the Likelihood Ratio Test. Multiple testing correction was applied with the Benjamini Hochberg False Discover Rate Correction (FDR) with a significance threshold of 0.05. Qiagen Ingenuity Pathway Analysis® was used for downstream pathway analysis.

## Results

**Fig 1:**
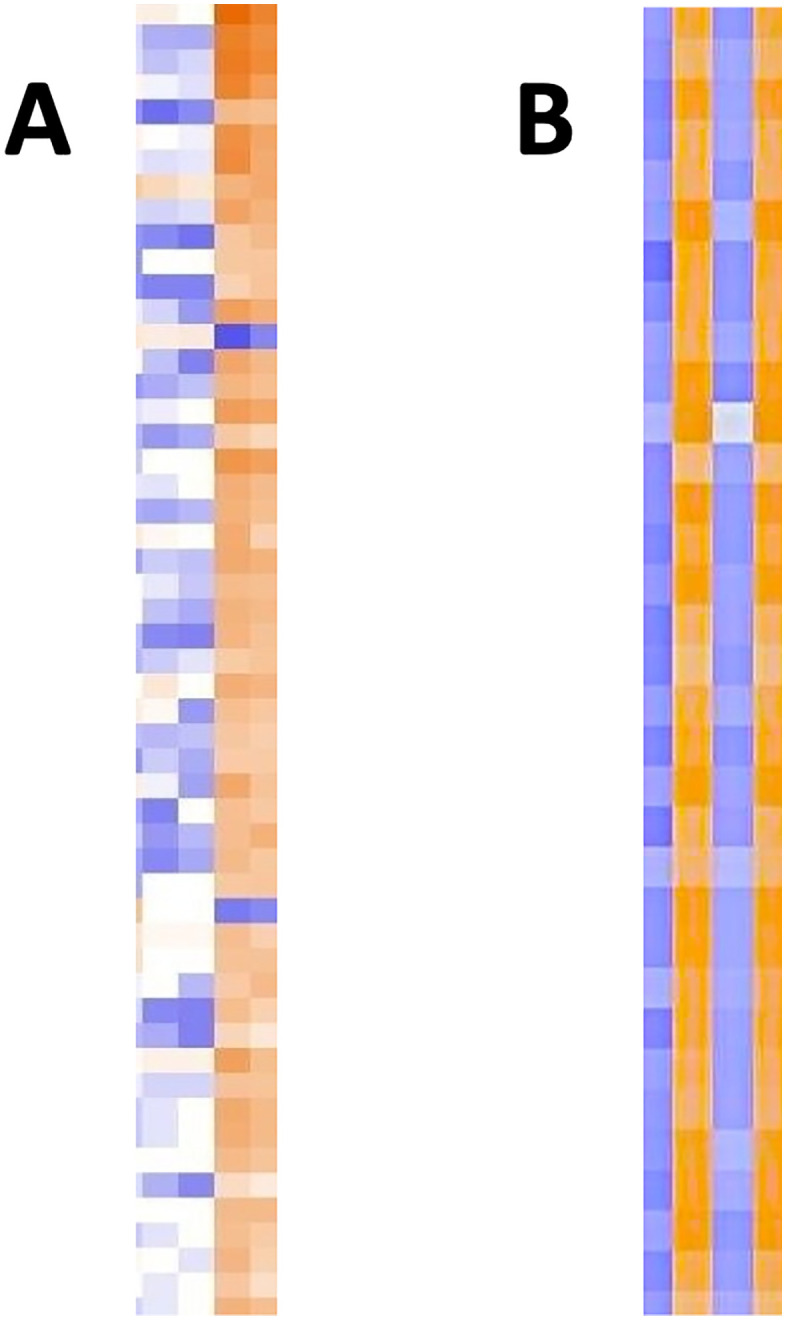
Transcription patterns following decapeptide exposure. A) RNA patterns for top 22 species following 1 hr exposure to decapeptide (62 pmole) for enteroids. Duplicate lanes – control lanes on left, decapeptide lanes on right. (Similar profiles were seen up to 4 hrs post-exposure.) B) RNA patterns for top 22 species 1 hr post-exposure to decapeptide (62 pmole) for HEK293 cells. Two sets of data showing control on left, exposure on right for each set.

After preliminary studies showed that only treatment with decapeptide #1 (mSAA) and not a scrambled sequence altered *E. coli* adherence, we emphasized the mSAA decapeptide alone in two different cell systems to address whether transcription was affected. We first asked whether exposing cultured enteroids to mSAA decapeptide led to transcriptional changes within one hour. Fig 1 A shows many changes and this pattern persisted for up to 4 hrs (data not shown). This initial observation led to studies using the technically simpler system of HEK293 monolayers and Fig 1 B also shows many transcriptional changes.

**Fig 2:**
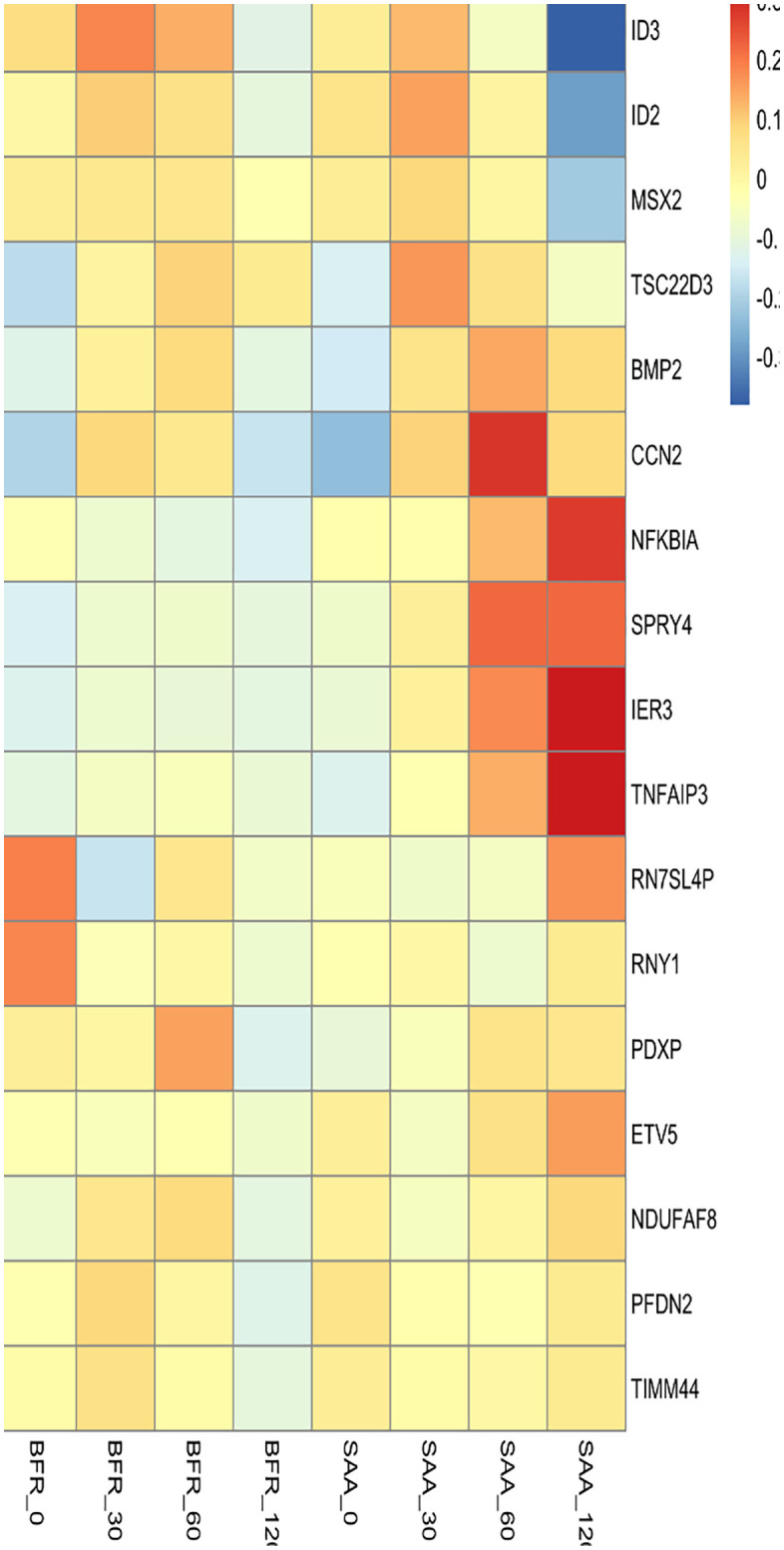
Heat map of transcription patterns using full-length SAA1 (4.3 mµmole) and HEK293 monolayers for up to 2 hrs. Summary of triplicate studies. Columns correspond to 0, 30, 60, 120 minute time points. The left 4 columns are controls; the right 4 are from exposed cells. Transcript identities indicated on the right. See also Table 1 for details of prominent changes.

Observations made using mSAA decapeptide were then extended to using full-length SAA and HEK293 monolayers (study in triplicate). 17 transcripts showed particularly prominent changes at 2 hours after SAA exposure. The heat map indicates both positively and negatively regulated transcripts (Fig 2).

**Table 1:** Prominent Transcript Changes for HEK293 cells exposed to full-length human SAA.

Studies performed in triplicate.				
Highest at 2 hours after SAA exposure				
Transcript	Map	pvalue	padjusted	Log2FoldChange
*NFKB1A*	14q13.2	2.56 X 10^–19^	2.13 X 10^–15^	3.58 X 10^−1^
*TNFA1P3*	6q23.3	1.77 X 10^–13^	1.44 X 10^−9^	5.65 X 10^−1^
*IER3*	6p21.33	4.11 X 10^−10^	3.77 X 10^−5^	6.18 X 10^−1^
*ETV5*	3q27.2	7.14 X 10^−6^	4.25 X 10^−3^	6.41 X 10^−2^
Highest at 1 hour after SAA exposure				
*CCN2*	6p23.2	4.99 X 10^−9^	1.45 X 10^−5^	4.23 X 10^−1^
*SPRY4*	5q31.3	7.30 X 10^−6^	4.99 X 10^−3^	4.75 X 10^−1^
Lowest at 2 hours after SAA exposure				
*ID3*	1p36.12	8.87 X 10^−7^	9.27 X 10^−4^	−2.22 X 10^−1^
*ID2*	2p25.1	4.11 X 10^−9^	1.45 X 10^−5^	−1.78 X 10^−1^

Table 1 shows p values for particularly notable changes in *NFKB1A, TNFAIP3, IER3, ETV5* and *SPRY4* transcripts (although *SPRY4* is highest at 1 hour).

The most prominent transcriptional response was for *NFKB1A* (14q13.2) which encodes IκBα which binds NF-κB heterodimers to keep them inactive and stably sequestered in the cytoplasm [19]. Phosphorylation of IκBα leads to its ubiquitinylation and subsequent degradation which then frees up NF-κB heterodimers to translocate into the nucleus. Interestingly, in the absence of IκBα there is a delay in stopping NF-κB activity (*e.g.,* termination of its response to TNF-α). Chen *et al* [20] showed a notable decrease in volume and size of cervical cancer tumors in HeLa cells following NFKB1A plasmid-mediated overexpression. Using a knock-out cell line, Hoffman *et al* [21] showed that IκBα provided strong negative feedback and fast turn-off of the NF-κB response. However, simple interpretation is complicated by the fact that NF-κB can induce IκBα synthesis and thus possibly establish an oscillating response system.

The second most prominent response was for *TNFA1P3* (6q23.3), a ubiquitin-editing enzyme (TNFα-induced protein 3, also called “A20”) that can inhibit NF-κB activity (and, hence, inflammation) by blocking NF-κB/Rel A movement to the nucleus. This limits duration of cellular response(s) after exposure to inflammatory signals such as TNF-α, IL-1β or pathogens via Toll-like receptors [22]. A20 contains both E3 ligase and deubiquitinase domains; in the TNFR pathway it can inactivate RIP1. In the LPS-responsive pathway A20 can disrupt the polyubiquitin chains from TRAF6 [23]. Mutations in A20 have been found in some individuals with chronic overactivation of innate immune cells, including rheumatoid arthritis, systemic lupus and lymphoproliferation [24]. It is important to note, however, that *TNFA1P3* is also a target gene for NF-κB and thus finding elevated transcription of both it and IκBα may reflect NF-κB stimulation as part of an oscillating circuit [25]. Our data do not exclude the possibility of SAA inducing NF-κB directly or indirectly although it was not among the most prominent transcripts.

*IER3* (6p21.33) – immediate early response protein 3 – helps protect cells from TNFα- or Fas-induced apoptosis. IER3 directly interacts with the C-terminal region of the RelA/p65 subunit of NFκB containing the transactivation domain and leading to reduced expression of anti-apoptotic target genes Bcl-2, Bcl-xL, cIAP1 and cIAP2 as well as increased cell sensitivity to apoptotic signals. It is expressed in pancreatic ductal adenocarcinoma and ovarian and other cancers and may be a clinical prognostic indicator. IER3 deficiency can be associated with prominent inflammation [26].

*ETV5* (3q27.2) encodes an RNA polymerase II-specific transcription factor variant (#5) enabling sequence-specific DNA-binding activity [27]. Prominently involved during oxidative stress, it has been implicated in growth of bladder, colorectal and ovarian cancers [28].

The *SPRY4* (5q31.3) transcript level peaks at 1 hour post SAA exposure. It encodes an inhibitor of the receptor-transduced mitogen-activated protein kinase (MAPK) signaling pathway and impairs formation of GTP-RAS [29] consistent with a possible role as part of a negative feedback loop [30]. It is upregulated in response to hypoxia and iron depletion [31]. SPRY4 may be associated with either decreased inflammatory change (*e.g.,* ROS expression) or proinflammatory changes, depending on the cell type and stimulation [32].

The *CCN2* (6p23.2) transcript, also prominent at 1 hour, encodes a mitogen related to pdgf and is related to cellular adhesion [33]. Also known as CTGF, it is involved in regeneration of alveolar epithelial type 2 cells following lung injury [34]. Interestingly, levels of both *SPRY4* and *CCN2* transcripts are slightly lower by the 2 hr point.

Downregulation of 2 transcripts of inhibitors of DNA binding are also noted. ID2 (2p25.1) inhibits functions of basic helix-loop-helix transcription factors. Similarly, ID3 (1p36.12), also a helix-loop-helix protein, can inhibit DNA binding of other factors and, hence, their transcriptional activity [35]. Both can form heterodimers [36,37].

*MSX2* encodes a transcriptional repressor and has been particularly notable in development where it is a member of the muscle segment homeobox gene family. It can function as a corepressor protein in several pathways, notably in Notch signaling [38].

## Discussion

Our findings widen the array of cell systems influenced by SAA beyond cells of the macrophage lineage. We report that exposing HEK293 cells to SAA leads to rapid, prominent transcription of genes involved in inflammation, in particular, several affecting nuclear transport of NF-κB and its transcriptional consequences.

We have sought to understand the molecular consequences of elevated serum SAA levels that are prominent in many biologic contexts including chronic inflammatory disorders, atherosclerosis, various cancers and perinatal health [1]. Without pathophysiologic clarity, previous reports have proposed that serum level(s) of SAA may be used to monitor several of these conditions although assays for it are somewhat cumbersome and the ”C-reactive protein” (CRP) often has been used as a surrogate.

Long-term consideration of the “biology” of SAA must consider its participation in the APR. This striking, stereotyped “primordial” reaction to various stimuli has been recognized for nearly a century. As noted above, SAA and CRP are the most prominent serum proteins during this process, quickly reaching peak levels and then returning to baseline. In chronic inflammatory conditions relatively high serum levels of both may persist, although their levels are generally not as high as during the APR. Although important 5’ regions of SAA gene structure as well as associated transcription factors have been identified (*e.g.,* [5,7–9]) it is unclear how the process resolves. At least for the APR model, the presumption has been that once the stimulus (-i) level falls, the process will resolve. By contrast, chronic elevations of SAA levels as seen in inflammatory conditions (*e.g.,* infections, arthritis, possibly neoplasia) and can lead to amyloid fibril formation – arise due to persistent cytokine (*e.g.,* IL-1, IL-6, TNF) stimulation of transcription.

Chronically elevated serum levels of SAA levels can be associated with deposition of fibrils derived from N-terminal fragments of SAA. These inert “amyloid” fibrils are recognized as the basis for “secondary” amyloidosis as encountered clinically and experimentally. Interestingly, the three-dimensional structure of the SAA monomer that, as noted, contains prominent α-helices [4] must be completely disrupted to form the prominent β-sheet domains of the fibrils [34]. Of longstanding interest in terms of pathology, formation of these virtually inert, potentially toxic (if only due to their physical bulk and interruption of organ integrity) fibrils begins intracellularly – the low pH of the lysosome enables structural reorganization.

**Fig 3:**
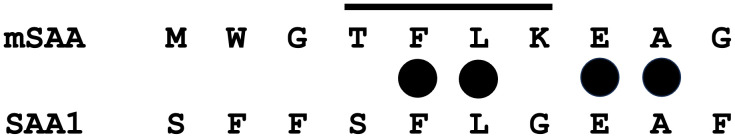
Comparison of N-terminal sequences for mSAA and SAA1. The TFLK sequence (overlined) is not present in human SAA1. The only correspondences for this region are indicated by the dots.

Multiple “receptors” have been proposed for SAA (see [1]) and more than one may be involved in the putative physiologic feedback system. Of particular interest in this regard are our findings that both the N-terminal decapeptide of mSAA and full-length human SAA can induce significant transcription. The “mSAA” decapeptide used here was defined in studies by McDonald *et al.* [17,18] as particularly involved in veterinary mastitis. These workers emphasized the T-F-L-K sequence as common to their isolates from several animals. This sequence is *not* present in circulating human SAA proteins. It is, however, encoded by a member of the human SAA gene family [39]. Although this gene is transcribed in humans, it contains a single nucleotide deletion that leads to loss of the corresponding mRNA [40]. Comparison of the mSAA decapeptide with the corresponding region of circulating human SAA shows relatively few conserved residues, in particular, the T-F-L-K sequence is absent (Fig 3).

Despite these differences, our data show that mSAA decapeptide exposure leads to impressive transcriptional responses in two, quite different, systems (enteroids and HEK293 cells) and full-length human SAA has similar effects in HEK293 cells. The 3-dimensional structure of SAA [4] shows that this decapeptide is part of an exposed α-helix on the surface of the monomer and, hence, could be directly exposed to surface receptor(s). Our data indicate that this short helical region can act alone (as seen for the decapeptide) for at least initial contact. AlphaFold predictions for both of these decameric regions show relatively few structural differences. Thus, a common aspect of this sequence (probably α-helical) is likely involved with cell interactions.

It is also of note that a fragment of SAA comprising amino acids 11−58 suppressed LPS-induced IL-1β, IL-6, and TNF-α in murine bone marrow-derived macrophages and improved C57BL/6 mouse survival after a lethal dose of LPS [41]. The same fragment induced IL-10 in a TLR-2 dependent but TLR-4 independent manner, suggesting an anti-inflammatory role for domains of SAA beyond the decapeptide studied here.

Interestingly, we did not find evidence for transcription mediated through the AHR receptor (as reported in AML cells [10]); specifically, no stimulation was found for either *IDO1* [10] or other genes known to be in the pathway (*e.g*., *CYPTB1*, *RELA* or *JUN* [42]). We did not address TH17 cell differentiation and IL-17 secretion [11]. It is likely that different cell types respond differently (or not at all) to SAA.

Using macrophages, Mohanty *et al*. [43] distinguished alternative roles for SAA depending on the physiologic conditions. In sterile inflammation SAA can interact with TLR4 to activate NF-κB and cytokine production while In infections SAA can interact with bacterial PAMPs, for example through opsinization [13], leading to bacterial clearance and immune cell recruitment.

Our finding these acute transcriptional sequelae of SAA exposure in cells beyond the macrophage lineage supports the hypothesis that members of this evolutionarily conserved protein family have an important role(s) in inflammation. By stimulating mRNA transcription for and synthesis of factors involved with regulating NF-κB (and, likely, other proteins) SAA may be important in regulating host defense. It is likely that SAA may, itself, be a physiologic mediator, providing feedback control of multiple chronic pathologic processes in different cells by modulating transcription. We therefore propose that SAA is involved in regulating basic inflammatory pathways. In fact, the high SAA levels seen in the APR may affect multiple cell types and be at least partially responsible for rapid resolution of the process.

The broad array of high SAA levels reported in many conditions could thus reflect physiologic attempts to reestablish homeostasis and, thus, oppose systemic consequences of inflammation, Infection, neoplasia and generalized stress. It has not escaped our notice that SAA (or a derived fragment such as the N-terminal decapeptide) may have therapeutic utility for reducing inflammation and its sequelae as well as in other proliferative and transcription-related processes.
